# An online survey on clinical characteristics of otologic symptoms linked to COVID-19 infection

**DOI:** 10.3389/fpubh.2023.1184262

**Published:** 2023-05-26

**Authors:** Linsui Wu, Hongyi Peng, Yufeng He, Ling Pu, Shixun Zhong

**Affiliations:** Department of Otolaryngology, The First Affiliated Hospital of Chongqing Medical University, Chongqing, China

**Keywords:** COVID-19, otologic symptoms, cross-sectional survey, clinical characteristics, pathological mechanisms

## Abstract

**Objective:**

To report the otologic symptoms that present in patients with COVID-19 infection and investigate the pathogenic characteristics during the period of the pandemic.

**Materials and methods:**

This cross-sectional descriptive study included participants with COVID-19 infection. COVID-19 infection was verified in these patients by nucleic acid test or antigen test. An online questionnaire was developed to analyze the association between the COVID-19 pandemic and the characteristics of otologic symptoms.

**Results:**

This study included 2,247 participants, of which nearly half had one or more otologic symptoms. The presents of otologic symptoms were associated with gender (OR = 1.575, *p* < 0.0001), age (OR = 0.972, *p* < 0.0001), and occupation (healthcare worker: *p* < 0.0001; personnel of enterprises or institutions: OR = 1.792, *p* < 0.0001; student: OR = 0.712, *p* < 0.044). The otologic symptoms following COVID-19 infection in order were vertigo (25.95%), tinnitus (19.05%), otalgia (19.00%), aural fullness (17.18%), hearing loss (11.62%), otorrhea (1.25%), and facial paralysis (0.27%).

**Conclusion:**

The present study shows that otologic symptoms are common among the COVID-19 infected participants and that these symptoms mostly recover spontaneously. During the corona-virus pandemic, the involvement of the cochleovestibular system and facial nerve should not be overlooked while treating the COVID-19 infected individuals.

## 1. Introduction

Almost 3 years have passed since the World Health Organization declared the coronavirus infection (COVID-19) a pandemic. Enormous progress has been made in the impact and response to life-threatening symptoms of COVID-19 across the lifespan (1). Current studies focus on clinical features following COVID-19 infection more in major organs such as lung and heart. However, the concern over otologic manifestations following COVID-19 infection is relatively limited (2–5).

Evidence suggests that hearing loss, tinnitus, vertigo, and facial palsy may work as a potential long-term sequela of COVID-19 reducing the quality of life and negatively affecting interpersonal communication and social life (6–8). Meanwhile, current findings raise the value of unexplained cochleovestibular symptoms during the pandemic, as these may be the only presenting symptoms indicating COVID-19 or partial (1). Therefore, identifying otologic symptoms is very critical.

Though many papers have reported audiovestibular symptoms or facial palsy associated with COVID-19 infection, the underlying pathological mechanisms of otologic symptoms are still unclear (9–11).

To investigate the otologic manifestations thoroughly during the COVID-19 pandemic and to analyze the potential predictive variables, this study investigates the onset, duration, and clinical outcomes of otologic symptoms in patients with COVID-19 infection during the pandemic period in China. To the best of our knowledge, this is an epidemiological survey on this issue with the largest sample size to date.

## 2. Materials and methods

### 2.1. Participants

This study included 2247 COVID-positive participants comprising 1,138 without otologic symptoms and 1,109 with otologic symptoms. The patients with COVID-19 infection verified with nucleic acid test or antigen test were recruited in the pandemic period from December 20, 2022 to January 10, 2023. Potential COVID-19-positive participants were approached through a social media application (WeChat). All participants were from 30 provinces in China. Table 1 shows the demographic characteristics of all participants with COVID-19.

**Table 1:** General statistics.

**Variables**	**Population**	**Without otologic symptoms**	**With otologic symptoms**	^*^P
	***N*** **(%)**	***N*** **(%)**	***N*** **(%)**	
	**2,247**	**1,138 (50.65)**	**1,109 (49.35)**	
Age (years), Mean (P_25_, P_75_)	36.0 (28.0,46.0)	38.0 (30.0,48.0)	35.0 (28.0,42.0)	**0.001**
**Gender**				
Males	770 (34.27)	443 (38.93)	327 (29.49)	**0.001**
Females	1477 (65.73)	695 (61.07)	782 (70.51)	
**Occupation**				
Health care worker	1076 (47.89)	573 (50.35)	503 (45.36)	**0.003**
Teacher	133 (5.92)	66 (5.80)	67 (6.04)	
Personnel of enterprises or institutions	306 (13.62)	122 (10.72)	184 (16.59)	
Student	207 (9.21)	105 (9.23)	102 (9.2)	
Farmer	35 (1.56)	20 (1.76)	15 (1.35)	
Soldier	30 (1.34)	11 (0.97)	19 (1.71)	
Freelance	185 (8.23)	103 (9.05)	82 (7.39)	
Others	275 (12.24)	138 (12.13)	137 (12.35)	
**Education level**				
Junior high school or below	99 (4.41)	53 (4.66)	46 (4.15)	0.121
High school	157 (6.99)	81 (7.12)	76 (6.85)	
Undergraduate degree	1505 (66.98)	734 (64.5)	771 (69.52)	
Graduate degree or above	486 (21.63)	270 (23.73)	216 (19.48)	
**Smoking history**				
No	2011 (89.5)	1016 (89.28)	995 (89.72)	0.733
Yes	236 (10.5)	122 (10.72)	114 (10.28)	
**Drinking history**				
No	1329 (59.15)	656 (57.64)	673 (60.69)	0.143
Yes	918 (40.85)	482 (42.36)	436 (39.31)	
**COVID-19 vaccination history**				
No	51 (2.27)	29 (2.55)	22 (1.98)	0.369
Yes	2196 (97.73)	1109 (97.45)	1087 (98.02)	
**Comorbidities**				
No	1922 (85.5)	974 (85.6)	948 (85.5)	0.943
Yes	325 (14.5)	164 (14.4)	161 (14.5)	
**Hypertension**				
No	2120 (94.3)	1059 (93.1)	1061 (95.7)	**0.007**
Yes	127 (5.7)	79 (6.9)	48 (4.3)	
**Diabetes**				
No	2206 (98.2)	1116 (98.1)	1090 (98.3)	0.697
Yes	41 (1.8)	22 (1.9)	19 (1.7)	

^*^P, Participants with otologic symptoms group vs. those without otologic symptoms group; The bold values indicate the *p* < 0.05 meaning statistically significant.

### 2.2. Study design

To investigate the characteristics of otologic symptoms in individuals with COVID-19 infection during the pandemic period, we conducted a descriptive and analytical cross-sectional study using an online anonymous questionnaire through Questionnaire Star (https://www.wjx.cn/) survey platform.

The study was conducted in accordance with the Declaration of Helsinki, and approved by the Clinical Research Ethics Committee of the First Affiliated Hospital of Chongqing Medical University (K2023-059). Data was managed anonymously.

Briefly, the questionnaire contained an introduction detailing the aim of the study and a statement of participant confidentiality and anonymity. Participants were required to complete the questionnaire consisting of three sections. Section 1 aimed to collect sociodemographic data (age, gender, occupation, education) and general health condition (vaccination, smoking history, drinking history, pre-existing chronic comorbidities). Then the questionnaire put forward a critical question as to whether they ever had any of the following otologic symptoms: otalgia, hearing loss, tinnitus, aural fullness, vertigo, dizziness, disequilibrium, otorrhea, and facial paralysis following COVID-19 infection. If the response was NOT, then the survey was over. If the response was YES, then continue to complete Section 2 designed to investigate general COVID-19 symptoms, including fever/chill, respiratory symptoms (nasal congestion, runny nose, cough, sore throat), systematic symptoms (asthenia, ache, diarrhea, poor appetite), and others (anosmia, dysgeusia). Otologic symptoms following COVID-19 infection were assessed in Section 3. Specifically, we asked participants if they had new otologic symptoms and the onset, duration, and clinical outcomes of these symptoms. Furthermore, participants with pre-existing otologic symptoms were asked if their symptoms deteriorated after contracting COVID-19. In addition, participants were asked if they took any medicine following the COVID-19 infection.

In our study, we included participants if they met the following inclusion criteria: (1) COVID-19 infection was verified by nucleic acid test or antigen test; (2) Participation in the study was voluntary. The incorrect and uncompleted questionnaires have been excluded.

### 2.3. Statistical analysis

SPSS 26.0 for Windows software (SPSS Inc., Chicago, Il, USA) was used for statistical analysis. Descriptive statistics included total numbers (N), percentages (%), median (Mean) and interquartile range (IQR). Single factor analysis was used with the chi-square test, Fisher's exact test, or the Mann–Whitney U test for predictor variables (demographics, comorbidities, clinical characteristics, and presentation). Logistic regression analysis was subsequently used to assess the associations between each significantly different variable and the outcome. Odds ratio (OR), *p*-value ( ≤ 0.05), and 95% confidence interval (CI) were used to identify any significant relationships among variables.

## 3. Results

### 3.1. Participant characteristics

We recruited 2,247 COVID-positive participants comprising 1,138 without otologic symptoms and 1,109 with otologic symptoms. Table 1 shows the demographic characteristics of all participants with COVID-19. The study included 34.27% males and 65.73% females with a median age of 36.0 years (IQR, 28.0 to 46.0 years). Among the individuals, healthcare workers accounted for the largest proportion (47.89%), and soldiers the least (1.34%). The vast majority of the participants were highly educated [with an undergraduate degree (66.98%), with a graduate degree or above (21.63%)], a few smoked (10.5%), more than half had no drinking history (59.15%), and almost all were vaccinated with the COVID-19 vaccine (97.73%). The most frequent comorbidities were hypertension (5.7%) and diabetes (1.8%).

Among these demographic and comorbidities variables, we found significant differences in age, gender, occupation, and incidence rate of hypertension between the participants without otologic symptoms and those with otologic symptoms. A subsequent binary logistic regression revealed that the COVID-19 participates with otologic symptoms were associated with age (OR = 0.972, 95%CI:0.963–0.981, *p* < 0.0001), gender (OR = 1.575, 95%CI:1.311–1.893, *p* < 0.0001), occupation (healthcare worker: *p* < 0.0001; personnel of enterprises or institutions: OR = 1.792, 95%CI:1.376–2.333, *p* < 0.0001; student: OR = 0.712,95%CI:0.511-0.991, *p* < 0.044) (Table 2).

**Table 2:** Multivariable regression analysis for otologic symptoms.

**Multivariable analysis**	**b**	**b standard error**	**Wald**	***P***	**OR**	**95%CI**
Age (years)	−0.029	0.005	38.1	**0.0001**	0.972	0.963–0.981
**Gender**						
Females	0.454	0.094	23.531	**0.0001**	1.575	1.311–1.893
**Occupation**						
Health care worker^*^			30.98	**0.0001**		
Teacher	0.185	0.188	0.967	0.325	1.203	0.832–1.739
Personnel of enterprises or institutions	0.583	0.135	18.781	**0.0001**	1.792	1.376–2.333
Student	−0.34	0.169	4.052	**0.044**	0.712	0.511–0.991
Farmer	0.115	0.353	0.106	0.745	1.122	0.561–2.243
Soldier	0.604	0.395	2.334	0.127	1.829	0.843–3.969
Freelance	−0.095	0.163	0.338	0.561	0.91	0.661–1.252
Others	0.181	0.138	1.725	0.189	1.199	0.915–1.571
Hypertension	−0.02	0.202	0.009	0.923	0.981	0.660–1.457

^*^Each occupation vs. health care worker respectively. The bold values indicate the *p* < 0.05 meaning statistically significant.

### 3.2. Otologic symptoms

**Figure 1:**
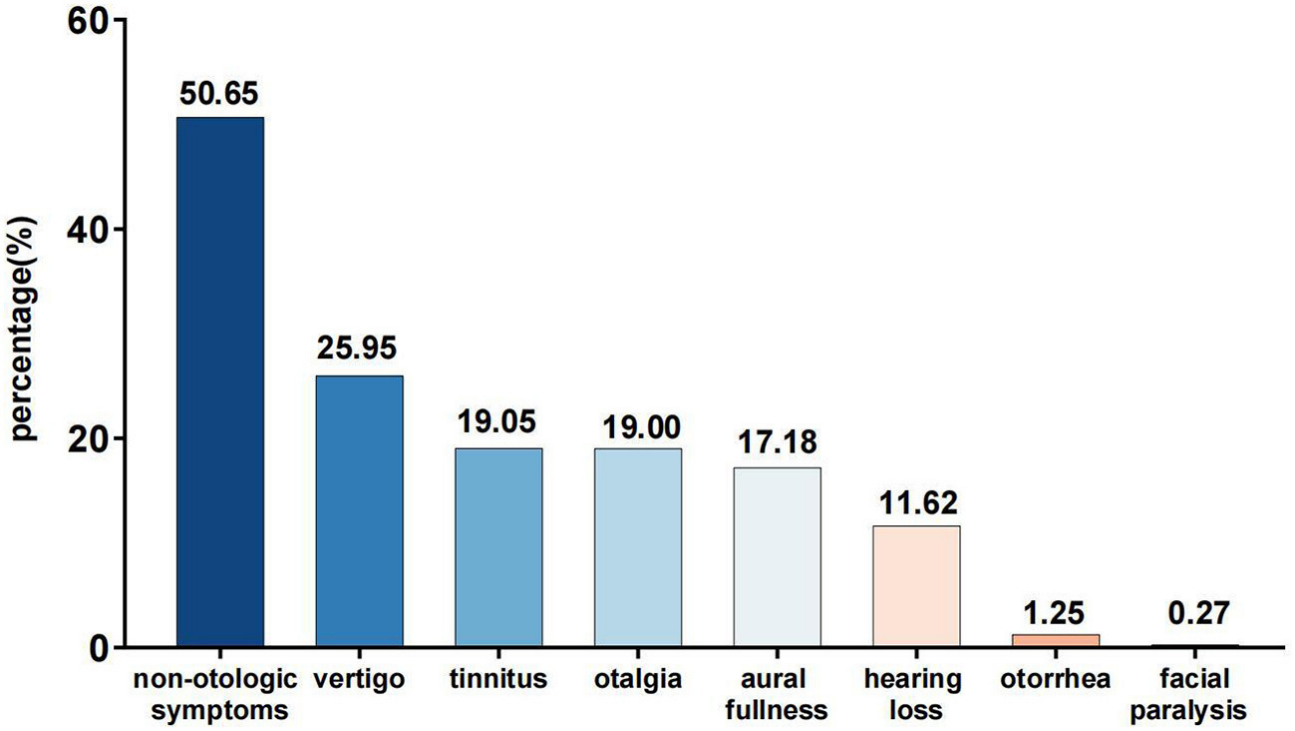
Otologic symptoms in the COVID-19 infected individuals.

Among all the COVID-19 infected participants, the number of participants complaining of vertigo, tinnitus, otalgia, aural fullness, hearing loss, otorrhea, and facial paralysis were 583 (25.95%), 428 (19.05%), 427 (19.00%), 386 (17.18%), 261 (11.62%), 28 (1.25%), 6 (0.27%), respectively (Figure 1).

The most common otologic symptom reported in the course of COVID-19 infection was vertigo. Of the 583 participants with vertigo (dizziness, disequilibrium), 401 (68.78%) reported dizziness, 222 (38.08%) reported vertigo attacks with body position change, 161 (27.62%) reported experiencing unstable standing and walking, 68 (11.66%) reported vertigo who felt spinning around. More than half patients had nausea (52.66%), 38.59% had sweating, and 21.27% had vomiting during vertigo attacks. Nearly half of these participants had occasional vertigo attacks (48.54%). There were 83.19% experiencing vertigo for the first time and 16.81% with previous vertigo disease. In 48.71% of participants, vertigo recovered completely, and in 40.65% partially (Table 3).

**Table 3:** Questionnaire data of the COVID-19 infected patients with vertigo.

**Survey question**	**Value(s)**
	***N*** **(%)**
	**583**
**Characteristics**	
Vertigo	68 (11.66)
Dizziness	401 (68.78)
Unstable standing and walking	161 (27.62)
Attack when a body position change	222 (38.08)
Others	32 (5.49)
**Accompanying symptoms**	
Nausea	307 (52.66)
Vomiting	124 (21.27)
Sweating	225 (38.59)
Others	146 (25.04)
**Severity**	
Occasionally	283 (48.54)
Intermittently, tolerable	197 (33.79)
Continuously, tolerable	81 (13.89)
Intolerable	22 (3.77)
**Vertigo history**	
No	485 (83.19)
Yes	98 (16.81)
**Medication**	
No	462 (79.25)
Yes	121 (20.75)
**Prognosis**	
Complete recovery	284 (48.71)
Partial recovery	237 (40.65)
Persistent	62 (10.63)

Tinnitus was the second most common otologic symptom. Our study found that 173 (40.42%) patients described their tinnitus as occasional, 162 (37.85%) as intermittent and tolerable, 86 (20.09%) as continuous and tolerable, and only 7 (1.64%) as intolerable. The quantities of the COVID-19 infected participants with tinnitus who described their tinnitus as low-frequency, high-frequency, and hard to say were similar. Most cases recovered completely or partially without taking medicines (Table 4).

**Table 4:** Questionnaire data of the COVID-19 infected patients with tinnitus.

**Survey question**	**Value(s)**
	***N*** **(%)**
	**428**
**Side**	
Unilateral	224 (52.34)
Bilateral	204 (47.66)
**Severity**	
Occasionally	173 (40.42)
Intermittently, tolerable	162 (37.85)
Continuously, tolerable	86 (20.09)
Intolerable	7 (1.64)
**Characteristics**	
Low-frequency	164 (38.32)
High-frequency	121 (28.27)
Hard to say	143 (33.41)
**Tinnitus history**	
No	241 (56.31)
Yes	187 (43.69)
**Medication**	
No	393 (91.82)
Yes	35 (8.18)
**Prognosis**	
Complete resolution	184 (42.99)
Partial recovery	179 (41.82)
Persistent	65 (15.19)

In our study, participants with unilateral otalgia (56.67%) were a little more than those with bilateral otalgia (43.33%). Otalgia usually tended to be intermittent (82.90%). The median score of visual analog scale used to describe the severity of otalgia was 4.0 (IQR, 3.0 to 6.0). Most cases did not take any medicine (Table 5).

**Table 5:** Questionnaire data of the COVID-19 infected patients with otalgia.

**Survey question**	**Value(s)**
	***N*** **(%)**
	**427**
**Side**	
Unilateral	242 (56.67)
Bilateral	185 (43.33)
**Severity** (0–10)	
Mean (P25, P75)	4.0 (3.0, 6.0)
**Characteristics**	
Intermittently	354 (82.9)
Continuously	73 (17.1)
**Medication**	
No	301 (70.49)
Yes	126 (29.51)

Aural fullness was found in more than one-third of all participants with otologic symptoms (34.81%). 155 (40.16%) described their aural fullness as occasional, 147 (38.08%) as intermittent and tolerable, 75 (19.43%) as continuous and tolerable, and only 9 (2.33%) as intolerable. Almost all cases recovered completely or partially without taking any medicine (Table 6).

**Table 6:** Questionnaire data of the COVID-19 infected patients with aural fullness.

**Survey question**	**Value(s)**
	***N*** **(%)**
	**386**
**Side**	
Unilateral	189 (48.96)
Bilateral	197 (51.04)
**Severity**	
Occasionally	155 (40.16)
Intermittently, tolerable	147 (38.08)
Continuously, tolerable	75 (19.43)
Intolerable	9 (2.33)
**Aural fullness history**	
No	298 (77.20)
Yes	88 (22.80)
**Medication**	
No	343 (88.86)
Yes	43 (11.14)
**Prognosis**	
Complete resolution	179 (46.37)
Partial recovery	155 (40.16)
Persistent	52 (13.47)

Hearing loss was reported by 11.62% of participants. The severity of hearing loss was estimated by patients based on their subjective feeling as mild (78.16%), medium (17.62%), and severe (4.21%). Most participants could not determine how long exactly this symptom had persisted. Furthermore, one unanticipated finding was that participants with bilateral hearing loss were more than those with unilateral hearing loss. 83.14% of participants didn't take any medication during the course of hearing loss. 26.82% of participants recovered completely, and 55.17% recovered partially (Table 7).

**Table 7:** Questionnaire data of the COVID-19 infected patients with hearing loss.

**Survey question**	**Value(s)**
	***N*** **(%)**
	**261**
**Side**	
Unilateral	109 (41.76)
Bilateral	152 (58.24)
**Severity**	
Mild	204 (78.16)
Medium	46 (17.62)
Severe	11 (4.21)
**Hearing loss history**	
No	195 (74.71)
Yes	66 (25.29)
**Medication**	
No	217 (83.14)
Yes	44 (16.86)
**Prognosis**	
Complete resolution	70 (26.82)
Partial recovery	144 (55.17)
Persistent	47 (18.01)

It was shown that 28 out of 2247 participants (1.25%) had otorrhea. 13 (46.43%) described their otorrhea as occasional, 7 (25.00%) as intermittent, and 8 (28.57%) as continuous. The proportion of the COVID-19 infected participants with unilateral otorrhea was much higher than those with bilateral otorrhea. A majority of these cases did not take medication and over half of the participants recovered completely or partially (Table 8).

**Table 8:** Questionnaire data of the COVID-19 infected patients with otorrhea.

**Survey question**	**Value(s)**
	***N*** **(%)**
	**28**
**Side**	
Unilateral	20 (71.43)
Bilateral	8 (28.57)
**Severity**	
Occasionally	13 (46.43)
Intermittently	7 (25.00)
Continuously	8 (28.57)
**Otitis media history**	
No	15 (53.57)
Yes	13 (46.43)
**Medication**	
No	24 (85.71)
Yes	4 (14.29)
**Prognosis**	
Complete resolution	5 (17.86)
Partial recovery	14 (50.00)
Persistent	9 (32.14)

The least common otologic symptom was facial paralysis (0.27%). Five out of 6 had previous history, but only 1 took medication. All of them recovered completely or partially (Table 9).

**Table 9:** Questionnaire data of the COVID-19 infected patients with facial paralysis.

**Survey question**	**Value(s)**
	***N*** **(%)**
	**6**
**Side**	
Unilateral	4 (66.67%)
Bilateral	2 (33.33%)
**Facial paralysis history**	
No	1 (16.67%)
Yes	5 (83.33%)
**Medication**	
No	5 (83.33%)
Yes	1 (16.67%)
**Prognosis**	
Complete resolution	1 (16.67%)
Partial recovery	5 (83.33%)
Persistent	0 (0.00%)

**Figure 2:**
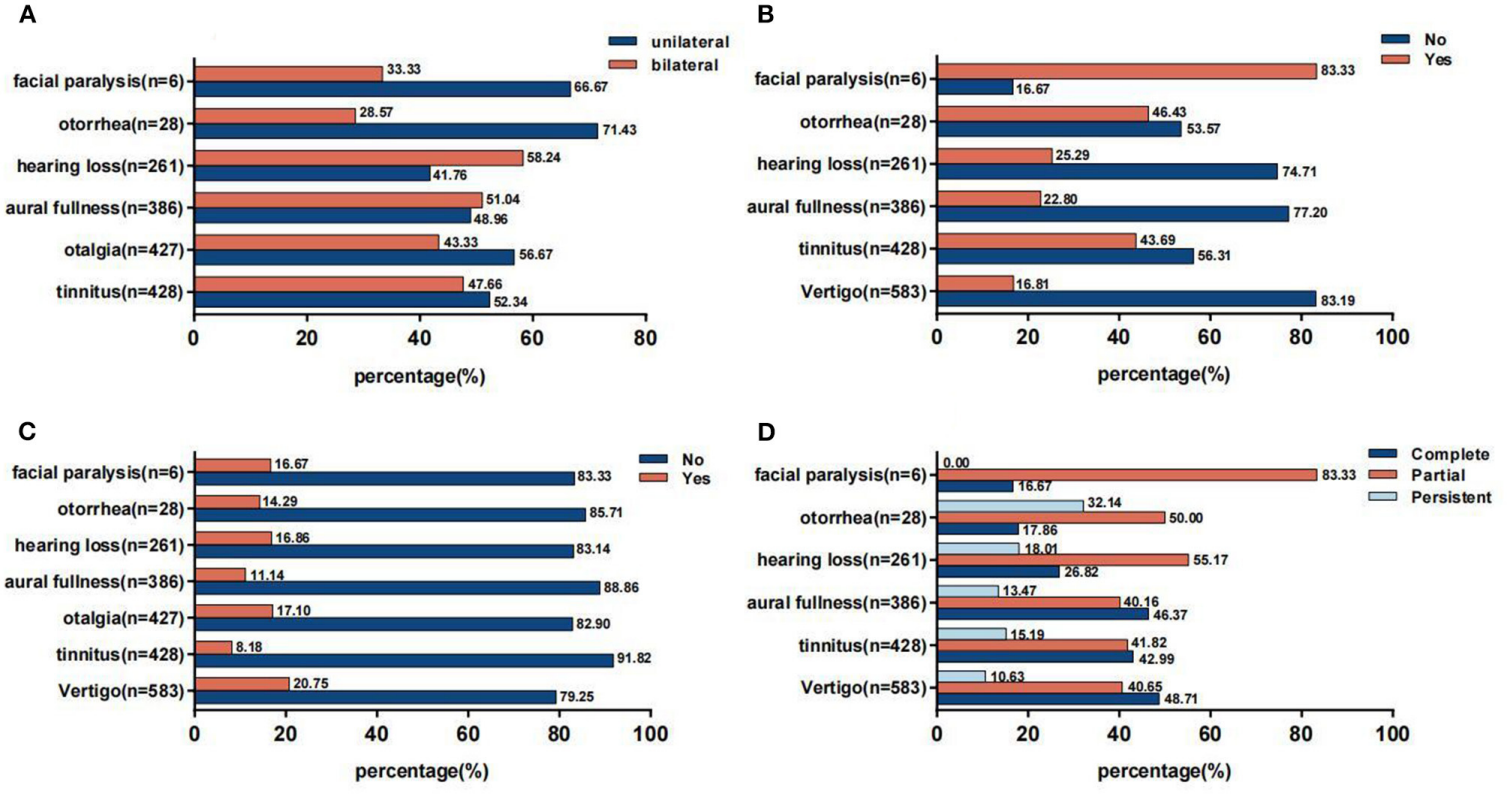
**(A)** Side of otologic symptoms. **(B)** Previous history of otologic symptoms. **(C)** Usage of medication for otologic symptoms. **(D)** Prognosis of otologic symptoms.

In general, there was not much difference in incidence between the unilateral and bilateral otologic symptoms except otorrhea and facial paralysis (Figure 2A). It was noteworthy that more than half suffered from new onset of otologic symptoms except those with facial paralysis (Figure 2B). In addition, the proportion of individuals taking no medications was overwhelmingly higher (Figure 2C). Furthermore, these symptoms mostly recovered partially or completely (Figure 2D).

### 3.3. General symptoms of COVID-19

**Figure 3:**
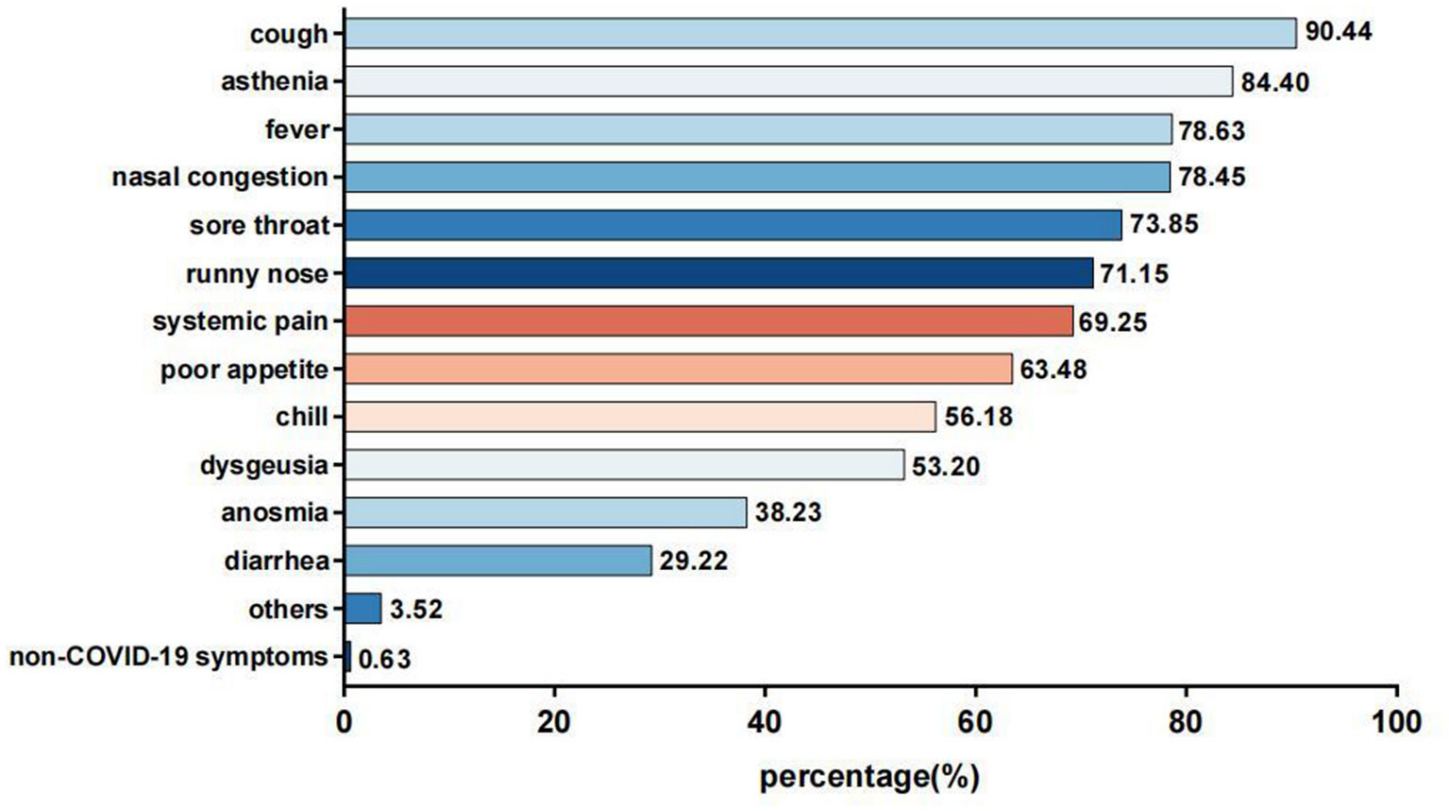
General symptoms of the COVID-19 infected with otologic symptoms.

Overall, 0.63% of the participants with otologic symptoms had no general symptoms. Similar to previous studies, the most frequently reported general symptoms were cough (90.44%), asthenia (84.40%), fever (78.63%), nasal congestion (78.45%), sore throat (73.85%), and runny nose (71.15%) (Figure 3).

## 4. Discussion

COVID-19 primarily infects the respiratory system (12). However, recent studies have demonstrated the involvement of not only major organs and systems like respiratory systems and cardiovascular system, but also cochleovestibular system and facial nerve (1, 13).

In this cross-sectional descriptive study, we found that nearly half the participants with positive nucleic acid test or antigen test of COVID-19 had one or more otologic symptoms through an online questionnaire survey during the COVID-19 pandemic period in China. Furthermore, occurrences of otologic symptoms were associated with gender, since female participants were significantly more affected than males. This finding is consistent with previous studies reporting that post-COVID-19 symptoms are more prevalent in women than in men (14, 15). Various theories have been proposed to explain the gender-related differences. For example, the higher expression level of the angiotensin-converting-enzyme-2 (ACE2) and lower level of pro-inflammatory cytokines (i.e., interleukin-6) in women after viral infections could explain their higher susceptibility to developing post-COVID otologic symptoms (16, 17). Additionally, unfavorable psychological factors, such as stress, sleep, anxiety, and depressive disorders, were observed to a greater extent in women and might also have an impact on perception of sensory symptoms such as dizziness (18).

Surprisingly, we found that the older adults were less likely to have otologic symptoms. Previous reports have shown that infected older individuals have more severe clinical symptoms and outcomes, and the association between age and symptoms/outcomes is often attributed to increased comorbidities in the older adults. Nonetheless, Almishaal et al. (19) did not find a statistically significant increase in audiovestibular symptoms among the old compared to younger participants. Moreover, our result was consistent with the study by Lechien et al. (3) who reported that young people more often manifested ENT symptoms.

In addition, the professions such as healthcare worker and personnel of enterprises or institutions were positive factors for otologic symptoms. This finding has extended our knowledge of the association between occupation and post-COVID-19 otologic symptoms. We speculated that this might be due to richer awareness of disease development from healthcare worker. In addition, the nature of the personnel of enterprises or institutions makes it easier for them to observe subtle changes in the body.

It has been well-known that vertigo may significantly affect the quality of life. Our study identified that vertigo was the most common otologic symptom. In the studies by Korkmaz et al. (20) and Zieba et al. (21) the incidence rates of vertigo were similar to ours (31.8%, 34%, and 25.95% respectively). There might be multiple explanations why individuals with COVID-19 infection experience vertigo (dizziness, disequilibrium). Firstly, the neuroinvasive and neurotropic properties of COVID-19 virus in the central and peripheral nervous systems have been reported (22–25). Recent studies have shown a high affinity of this virus for angiotensin-converting enzyme 2 (ACE2), which is frequently found in the nervous system and nasal mucosa (26, 27). COVID-19 can be transferred through the olfactory nerve and bulb to the central nervous system directly, or through viremia (24, 28). Secondly, novel coronavirus can directly infect the human inner ear and cochleovestibular nerve because human inner ear tissue co-expresses the ACE2 receptor for novel coronavirus (29). Furthermore, an autoimmune-mediated mechanism has also been proposed as a potential mechanism. In severe cases of COVID-19 infection, an autoimmune-mediated process causes an uncontrolled viral replication and an exaggerated systemic response leading to an increase of pro-inflammatory cytokine levels (i.e., cytokine storm) which may constitute a potential source of damage for many body organs including the inner ear (30–32). Vascular pathologies were also proposed as a potential mechanism for COVID-19-mediated vestibular manifestations given the evidence that a significant proportion of COVID-19 patients develop coagulation abnormalities (33, 34). It is worth of note that these are the mechanisms that not only may cause vertigo but also may cause other otologic symptoms such as tinnitus, otalgia, and hearing loss. It is important to highlight that factors other than the primary infection itself can generate vertigo symptoms. Studies have revealed a significant contribution of body weakness and fatigue resulting from metabolic and nutritional dysfunctions to the manifestation of vestibular symptoms during the acute phase of COVID-19 infection (19, 35).

Tinnitus was the second most common otologic symptom shown in the present study. It is unclear whether tinnitus is directly related to COVID-19 infection or not. Additionally, as stated in literatures, the relationship between COVID-19 and the onset of tinnitus or worsening of the preexisting tinnitus may depend on the negative effects of stress and anxiety generated by the pandemic process (36, 37). Our result falls within previously reported estimates ranging from 0.35% to as high as 35% (16). In addition, our study identified that nearly half participants with tinnitus presented worsening tinnitus, which was consistent with previous study by Xia et al. (38) who reported an increase in tinnitus severity during the pandemic.

We investigated otalgia following COVID-19 infection and found a similar incidence rate with that of tinnitus. The majority of sufferers presented with mild to moderate intermittent otalgia. Most patients with otalgia presented pain around the ear or in the ear canal without local redness, swelling, or heat. We speculate it as neuropathic pain due to direct involvement of trigeminal nerve and greater auricular nerve by virus (39). Indeed, certain aural diseases such as otitis media can also cause otalgia (39, 40).

Aural fullness is a recognized classical symptom of transient Eustachian tube dysfunction which may be triggered by many causes, the most common being upper respiratory viral infections (41). Indeed, as the cellular receptor for the COVID-19, ACE2 was detected in the Eustachian tube of mice and in the autopsy of the middle ear tissues of COVID-19 positive decedents, which indicates that these structures are likely susceptible to COVID-19 infection leading to aural fullness (42, 43).

Hearing loss following COVID-19 infection may be sensorineural, conductive, or mixed. Classification of hearing loss in this study was not available since audiometry is not possible for an online survey. A growing body of evidence suggests that patients with COVID-19 are at risk of developing sudden sensorineural hearing loss (SSNHL) (44, 45). The incidence of hearing loss (11.62%) in our study was consistent with a retrospective observational study, which showed that over 10% of COVID-19 patients with self-reported chemosensory loss complained of hearing loss (46). In contrast, the prevalence was higher than those in some earlier systematic reviews reporting a prevalence of about 5.08%−8.7% (13, 47). The precise reason for SNHL post COVID-19 infection is currently unknown yet, but recent studies have indicated the importance of endothelial dysfunction and micro-thrombosis (48). Also, the virus is associated with the increased activation of immune system (49, 50). It should be noted that the majority of the extant research emphasizes that viral infection of the cochlea nerve or central nervous system is one of the more common presumed etiologies (51, 52). In addition, there could be conductive deafness due to middle ear diseases such as otitis media and Eustachian tube dysfunction resulting from COVID-19 infection.

Few studies investigate otorrhea among COVID-19 infected individuals in detail, and only a few cases with otitis media have been reported. In our study, we found that the prevalence of otorrhea is much lower than the symptoms mentioned earlier. Of these participants, nearly half suffered from otitis media recurrence. A possible explanation for this might be that COVID-19 infects the middle ear directly through the ACE2. Moreover, some studies identified COVID-19 virus in mastoid or middle ear (43, 53). After being infected by COVID-19, on the one hand, the patients may suffer from weakened immunity that tends to result in otitis media. On the other hand, the middle ear may experience secondary bacterial infection, which deteriorates the existed otitis media.

Last but not least, some researches have reported that COVID-19 may cause facial nerve palsy (54, 55). Mehrdad Estakh et al. (11) have shown that there is enough evidence suggesting that patients with COVID-19 infection may present with facial palsy as the initial clinical manifestation. According to recent studies, the COVID-19 virus may damage facial nerve function by direct toxic effects on the nerve or by increasing hypercoagulopathy (23). Increased deterioration of nerve function can occur due to direct viral damage or an autoimmune event that can trigger a boost in inflammation of the nerve (56). For instance, facial palsy was regarded as neuronal damage secondary to severe complications like Guillain-Barré Syndrome (GBS) (57). Previous studies have shown combined facial and trigeminal nerve palsy after COVID-19 infection (58). Nevertheless, the incidence rate of facial palsy is very low. We found that only 6/2247 (0.27%) patients had facial paralysis, of which 5 out of 6 had previous history. Therefore, further studies are needed to investigate the potential mechanism of facial palsy following COVID-19 infection. Physicians, however, should keep undoubtedly in mind the likelihood of facial palsy post COVID-19 infection and treat it accordingly.

A key superiority of our study is that we recruited participants during the acute phase of COVID-19 infection with a large sample size. Furthermore, our questionnaire covers common otologic symptoms and thus enables a comprehensive analysis of the correlation between COVID-19 infection and otologic symptoms. Nevertheless, there are a few limitations that need to be considered in this study. Firstly, the data collected were self-reported by patients via the social media application WeChat without objective diagnostic and audiological tests. Secondly, the use of a single social media platform may result in sampling bias since some older individuals are less likely to complete the online questionnaire. Thirdly, the vast majority of questionnaires were filled out in a few days after COVID-19 infection. Further studies are needed to investigate the long-term impact of COVID-19 infection on ear.

## 5. Conclusion

Our study shows that otologic symptoms are common among the COVID-19 infected individuals during the acute phase of the pandemic period, and that these symptoms mostly appear to recover spontaneously. However, the true prevalence of involvement of the cochleovestibular system and facial nerve in COVID-19 patients around the world is unknown. Given that most of the studies are from a single institution with a small sample size, the published data must be interpreted with caution. In addition, more comprehensive otologic tests are required to further elucidate the pathogenesis of the underlying dysfunctions of the cochleovestibular system and facial nerve after COVID-19 infection.

## Data availability statement

The raw data supporting the conclusions of this article will be made available by the authors, without undue reservation.

## Ethics statement

The studies involving human participants were reviewed and approved by the Clinical Research Ethics Committee of the First Affiliated Hospital of Chongqing Medical University (K2023-059). Written informed consent to participate in this study was provided by the participants' legal guardian/next of kin.

## Author contributions

SZ and LW contributed to the design, analysis, and writing of the manuscript. HP and YH participated in conducting the study and supervised data acquisition. LP contributed to gathering data and drafting of tables and figures. All authors have read and agreed to the published version of the manuscript.

## References

[1] Khoza-Shangase K. Cochleovestibular findings linked to COVID-19: a scoping review for clinical care planning in South Africa. S Afr J Commun Disord. (2022) 69:e1–e12. 10.4102/sajcd.v69i2.899 36073075 PMC9452924

[2] Fotuhi M Mian A Meysami S Raji CA. Neurobiology of COVID-19. J Alzheimer's Dis JAD. (2020) 76:3–19. 10.3233/jad-200581 32538857 PMC7660990

[3] Lechien JR Chiesa-Estomba CM Place S Van Laethem Y Cabaraux P Mat Q. Clinical and epidemiological characteristics of 1420 European patients with mild-to-moderate coronavirus disease 2019. J Intern Med. (2020) 288:335–44. 10.1111/joim.13089 32352202 PMC7267446

[4] Munro KJ Uus K Almufarrij I Chaudhuri N Yioe V. Persistent self-reported changes in hearing and tinnitus in post-hospitalisation COVID-19 cases. Int J Audiol. (2020) 59:889–90. 10.1080/14992027.2020.1798519 32735466

[5] Dusan M Milan S Nikola D. COVID-19 caused hearing loss. Eur Arch Otorhinolaryngol. (2022) 279:2363–72. 10.1007/s00405-021-06951-x 34235578 PMC8263317

[6] Pazdro-Zastawny K Dorobisz K Misiak P Kruk-Krzemień A Zatoński T. Vestibular disorders in patients after COVID-19 infection. Front Neurol. (2022) 13:956515. 10.3389/fneur.2022.956515 36203969 PMC9531925

[7] Al-Ani RM. Ear, nose, and throat manifestations of COVID-19 and its vaccines. World J Clin Cases. (2022) 10:8808–15. 10.12998/wjcc.v10.i25.8808 36157654 PMC9477042

[8] Haider HF Szczepek AJ. Editorial: neurotological consequences of long COVID. Front Neurol. (2022) 13:1087896. 10.3389/fneur.2022.1087896 36479046 PMC9720380

[9] De Luca P Scarpa A Ralli M Tassone D Simone M De Campora L. Auditory disturbances and SARS-CoV-2 infection: brain inflammation or cochlear affection? Systematic review and discussion of potential pathogenesis. Front Neurol. (2021) 12:707207. 10.3389/fneur.2021.707207 34421805 PMC8373381

[10] Ong KMC Cruz TLG. Otologic and vestibular symptoms in COVID-19: a scoping review. World J Otorhinolaryngol Head Neck Surg. (2022) 8:287–96. 10.1002/wjo2.57 35599837 PMC9111077

[11] Estakhr M Tabrizi R Ghotbi Z Shahabi S Habibzadeh A Bashi A. Is facial nerve palsy an early manifestation of COVID-19? A literature review. Am J Med Sci. (2022) 364:264–73. 10.1016/j.amjms.2022.04.010 35429449 PMC9007824

[12] Yuki K Fujiogi M Koutsogiannaki S. COVID-19 pathophysiology: a review. Clin Immunol. (2020) 215:108427. 10.1016/j.clim.2020.108427 32325252 PMC7169933

[13] Almishaal AA. Comparative study of audiovestibular symptoms between early and late variants of COVID-19. Audiol Res. (2022) 12:680–95. 10.3390/audiolres12060065 36546906 PMC9774134

[14] Iqbal FM Lam K Sounderajah V Clarke JM Ashrafian H Darzi A. Characteristics and predictors of acute and chronic post-COVID syndrome: a systematic review and meta-analysis. EClinicalMedicine. (2021) 36:100899. 10.1016/j.eclinm.2021.100899 34036253 PMC8141371

[15] Yong SJ. Long COVID or post-COVID-19 syndrome: putative pathophysiology, risk factors, and treatments. Infect Dis. (2021) 53:737–54. 10.1080/23744235.2021.1924397 34024217 PMC8146298

[16] Fernández-de-Las-Peñas C Martín-Guerrero JD Pellicer-Valero ÓJ Navarro-Pardo E Gómez-Mayordomo V Cuadrado ML. Female sex is a risk factor associated with long-term post-COVID related-symptoms but not with COVID-19 symptoms: the long-covid-exp-cm multicenter study. J Clin Med. (2022) 11:413. 10.3390/jcm11020413 35054108 PMC8778106

[17] Ortona E Buonsenso D Carfi A Malorni W. Long COVID: an estrogen-associated autoimmune disease? Cell Death Discovery. (2021) 7:77. 10.1038/s41420-021-00464-6 33850105 PMC8042352

[18] Salari N Hosseinian-Far A Jalali R Vaisi-Raygani A Rasoulpoor S Mohammadi M. Prevalence of stress, anxiety, depression among the general population during the COVID-19 pandemic: a systematic review and meta-analysis. Global Health. (2020) 16:57. 10.1186/s12992-020-00589-w 32631403 PMC7338126

[19] Almishaal AA Alrushaidan AA. Short- and long-term self-reported audiovestibular symptoms of SARS-CoV-2 infection in hospitalized and nonhospitalized patients. Audiol Neurootol. (2022) 27:297–311. 10.1159/000521963 35240596 PMC9059062

[20] Özçelik Korkmaz M Eǧilmez OK Özçelik MA Güven M. Otolaryngological manifestations of hospitalised patients with confirmed COVID-19 infection. Eur Arch Otorhinolaryngol. (2021) 278:1675–85. 10.1007/s00405-020-06396-8 33011957 PMC7532931

[21] Zieba N Lisowska G Dadok A Kaczmarek J Stryjewska-Makuch G Misiołek M. Frequency and severity of ear-nose-throat (ENT) symptoms during COVID-19 infection. Medicina. (2022) 58:623. 10.3390/medicina58050623 35630040 PMC9143391

[22] Sahin AR. 2019 novel coronavirus (COVID-19) outbreak: a review of the current literature. Eur J Med Oncol. (2020) 3:12220. 10.14744/ejmo.2020.1222033078061

[23] Desforges M Le Coupanec A Dubeau P Bourgouin A Lajoie L Dubé M. Human Coronaviruses and other respiratory viruses: underestimated opportunistic pathogens of the central nervous system? Viruses. (2019) 12:14. 10.3390/v12010014 31861926 PMC7020001

[24] Li YC Bai WZ Hashikawa T. The neuroinvasive potential of SARS-CoV2 may play a role in the respiratory failure of COVID-19 patients. J Med Virol. (2020) 92:552–5. 10.1002/jmv.25728 32104915 PMC7228394

[25] Paybast S Gorji R Mavandadi S. Guillain-Barré syndrome as a neurological complication of novel COVID-19 infection: a case report and review of the literature. Neurologist. (2020) 25:101–3. 10.1097/nrl.0000000000000291 32618839 PMC7363390

[26] Egilmez OK Gündogan ME Yilmaz MS Güven M. Can COVID-19 cause peripheral facial nerve palsy? SN Comprehens Clin Med. (2021) 3:1707–13. 10.1007/s42399-021-00967-4 34056546 PMC8140315

[27] Brevini T Maes M Webb GJ John BV Fuchs CD Buescher G. FXR inhibition may protect from SARS-CoV-2 infection by reducing ACE2. Nature. (2022) 3:5594. 10.1038/s41586-022-05594-0 36470304 PMC9977684

[28] Wrapp D Wang N Corbett KS Goldsmith JA Hsieh CL Abiona O. Cryo-EM structure of the 2019-nCoV spike in the prefusion conformation. Science. (2020) 367:1260–3. 10.1126/science.abb2507 32075877 PMC7164637

[29] Jeong M Ocwieja KE Han D Wackym PA Zhang Y Brown A. Direct SARS-CoV-2 infection of the human inner ear may underlie COVID-19-associated audiovestibular dysfunction. Commun Med. (2021) 1:44. 10.1038/s43856-021-00044-w 34870285 PMC8633908

[30] Degen C Lenarz T Willenborg K. Acute profound sensorineural hearing loss after COVID-19 pneumonia. Mayo Clin Proceed. (2020) 95:1801–3. 10.1016/j.mayocp.2020.05.034 32753155 PMC7275185

[31] Koumpa FS Forde CT Manjaly JG. Sudden irreversible hearing loss post COVID-19. BMJ Case Reports. (2020) 13:8419. 10.1136/bcr-2020-238419 33051251 PMC7554505

[32] Alves de. Sousa F, Pinto Costa R, Xará S, Nóbrega Pinto A, Almeida ESC. SARS-CoV-2 and hearing: An audiometric analysis of COVID-19 hospitalized patients. J Otol. (2021) 16:158–64. 10.1016/j.joto.2021.01.005 33558808 PMC7857034

[33] Levi M Thachil J Iba T Levy JH. Coagulation abnormalities and thrombosis in patients with COVID-19. Lancet Haematol. (2020) 7:e438–e40. 10.1016/s2352-3026(20)30145-9 32407672 PMC7213964

[34] Mao L Jin H Wang M Hu Y Chen S He Q. Neurologic manifestations of hospitalized patients with coronavirus disease 2019 in Wuhan, China. JAMA Neurol. (2020) 77:683–90. 10.1001/jamaneurol.2020.1127 32275288 PMC7149362

[35] Oates CP Turagam MK Musikantow D Chu E Shivamurthy P Lampert J. Syncope and presyncope in patients with COVID-19. Pacing Clin Electrophysiol. (2020) 43:1139–48. 10.1111/pace.14047 32840325 PMC7461520

[36] Beukes E Ulep AJ Eubank T Manchaiah V. The impact of COVID-19 and the pandemic on tinnitus: a systematic review. J Clin Med. (2021) 10:2763. 10.3390/jcm10132763 34201831 PMC8268057

[37] Saunders GH Beukes E Uus K Armitage CJ Kelly J Munro KJ. Shedding light on SARS-CoV-2, COVID-19, COVID-19 vaccination, and auditory symptoms: causality or spurious conjunction? Front Public Health. (2022) 10:837513. 10.3389/fpubh.2022.837513 35296050 PMC8919951

[38] Xia L He G Feng Y Yu X Zhao X Chen Z. COVID-19 associated anxiety enhances tinnitus. medRxiv. (2020) 3:2020.07.02.20145532. 10.1101/2020.07.02.20145532 33544744 PMC7864409

[39] Li Z Liu T Yang N Han D Mi X Li Y. Neurological manifestations of patients with COVID-19: potential routes of SARS-CoV-2 neuroinvasion from the periphery to the brain. Front Med. (2020) 14:533–41. 10.1007/s11684-020-0786-5 32367431 PMC7197033

[40] Meinhardt J Radke J Dittmayer C Franz J Thomas C Mothes R. Olfactory transmucosal SARS-CoV-2 invasion as a port of central nervous system entry in individuals with COVID-19. Nat Neurosci. (2021) 24:168–75. 10.1038/s41593-020-00758-5 33257876

[41] Park MS Lee HY Kang HM Ryu EW Lee SK Yeo SG. Clinical manifestations of aural fullness. Yonsei Med J. (2012) 53:985–91. 10.3349/ymj.2012.53.5.985 22869482 PMC3423854

[42] Uranaka T Kashio A Ueha R Sato T Bing H Ying G. Expression of ACE2, TMPRSS2, and furin in mouse ear tissue, and the implications for SARS-CoV-2 infection. Laryngoscope. (2021) 131:E2013–e7. 10.1002/lary.29324 33296096

[43] Frazier KM Hooper JE Mostafa HH Stewart CM. SARS-CoV-2 virus isolated from the mastoid and middle ear: implications for COVID-19 precautions during ear surgery. JAMA Otolaryngol Head Neck Surg. (2020) 146:964–6. 10.1001/jamaoto.2020.1922 32701126 PMC7378866

[44] Kilic O Kalcioglu MT Cag Y Tuysuz O Pektas E Caskurlu H. Could sudden sensorineural hearing loss be the sole manifestation of COVID-19? An investigation into SARS-CoV-2 in the etiology of sudden sensorineural hearing loss. Int J Infect Dis IJID Pub Int Soc Infect Diseases. (2020) 97:208–11. 10.1016/j.ijid.2020.06.023 32535294 PMC7289736

[45] Meng X Wang J Sun J Zhu K. COVID-19 and sudden sensorineural hearing loss: a systematic review. Front Neurol. (2022) 13:883749. 10.3389/fneur.2022.883749 35572936 PMC9096262

[46] Thrane JF Britze A Fjaeldstad AW. Incidence and duration of self-reported hearing loss and tinnitus in a cohort of COVID-19 patients with sudden chemosensory loss: a STROBE observational study. Eur Ann Otorhinolaryngol Head Neck Dis. (2022) 139:125–8. 10.1016/j.anorl.2021.07.012 34602376 PMC8482223

[47] Almufarrij I Uus K Munro KJ. Does coronavirus affect the audio-vestibular system? A rapid systematic review. Int J Audiol. (2020) 59:487–91. 10.1080/14992027.2020.1776406 32530326

[48] Kumar Swain S Ranjan Pani S. Incidence of hearing loss in COVID-19 patients: a COVID hospital-based study in the Eastern part of India. Int J Curr Res Rev. (2021) 13:103–7. 10.31782/ijcrr.2021.13329

[49] McFadyen JD Stevens H Peter K. The emerging threat of (Micro)thrombosis in COVID-19 and its therapeutic implications. Circ Res. (2020) 127:571–87. 10.1161/circresaha.120.317447 32586214 PMC7386875

[50] Delgado-Roche L Mesta F. Oxidative stress as key player in severe acute respiratory syndrome coronavirus (SARS-CoV) infection. Arch Med Res. (2020) 51:384–7. 10.1016/j.arcmed.2020.04.019 32402576 PMC7190501

[51] Nile SH Nile A Qiu J Li L Jia X Kai G. COVID-19: pathogenesis, cytokine storm and therapeutic potential of interferons. Cytokine Growth Factor Rev. (2020) 53:66–70. 10.1016/j.cytogfr.2020.05.002 32418715 PMC7204669

[52] Chen X Fu YY Zhang TY. Role of viral infection in sudden hearing loss. J Int Med Res. (2019) 47:2865–72. 10.1177/0300060519847860 31130031 PMC6683896

[53] Mohan S Workman A Barshak M Welling DB Abdul-Aziz D. Considerations in management of acute otitis media in the COVID-19 era. Ann Otol Rhinol Laryngol. (2021) 130:520–7. 10.1177/0003489420958443 32911957

[54] Turki A Abbas KS Makram AM Elfert M Elmarabea M El-Shahat NA. Epidemiology, clinical features, and treatment modalities of facial nerve palsy in COVID-19 patients: a systematic review. Acta Neurol Belg. (2022) 122:1419–32. 10.1007/s13760-022-02026-8 35917018 PMC9345018

[55] Khurshid A Khurshid M Sohail A Raza IM Ahsan MK Alam Shah MUF. Facial palsy as a manifestation of COVID-19: a systematic review of cases. Health science reports. (2022) 5:e887. 10.1002/hsr2.887 36320650 PMC9616168

[56] Lima MA Silva MTT Soares CN Coutinho R Oliveira HS Afonso L. Peripheral facial nerve palsy associated with COVID-19. J Neurovirol. (2020) 5 26:941–4. 10.1007/s13365-020-00912-6 33006717 PMC7531061

[57] Namavarian A Eid A Ziai H Cheng EY Enepekides D. Facial nerve paralysis and COVID-19: a systematic review. Laryngoscope. 25:3033. (2022). 10.1002/lary.30333 35938708 PMC9538897

[58] Finsterer J Scorza FA Scorza C Fiorini A. COVID-19 associated cranial nerve neuropathy: a systematic review. Bosnian J Basic Med Sci. (2022) 22:39–45. 10.17305/bjbms.2021.6341 34392827 PMC8860318

